# RNA Degradation in Pluripotent Stem Cells: Mechanisms, Crosstalk, and Fate Regulation

**DOI:** 10.3390/cells14201634

**Published:** 2025-10-20

**Authors:** Seunghwa Jeong, Myunggeun Oh, Jaeil Han, Seung-Kyoon Kim

**Affiliations:** 1 Department of Convergent Bioscience and Informatics, Graduate School of Biological Sciences, Chungnam National University, Daejeon 34134, Republic of Korea; 2 Department of Microbiology and Molecular Biology, Chungnam National University, Daejeon 34134, Republic of Korea

**Keywords:** pluripotency stem cell, RNA degradation, post-transcriptional regulation, RNA modifications, degradome dynamics

## Abstract

Pluripotent stem cells (PSCs) exhibit remarkable self-renewal capacity and differentiation potential, necessitating tight regulation of gene expression at both transcriptional and post-transcriptional levels. Among post-transcriptional mechanisms, RNA turnover and degradation together play pivotal roles in maintaining transcriptome homeostasis and controlling RNA stability. RNA degradation plays a pivotal role in determining transcript stability for both messenger RNAs (mRNAs) and non-coding RNAs (ncRNAs), thereby influencing cell identity and fate transitions. The core RNA decay machinery, which includes exonucleases, decapping complexes, RNA helicases, and the RNA exosome, ensures timely and selective decay of transcripts. In addition, RNA modifications such as 5′ capping and N6-methyladenosine (m^6^A) further modulate RNA stability, contributing to the fine-tuning of gene regulatory networks essential for maintaining PSC states. Recent single-cell and multi-omics studies have revealed that RNA degradation exhibits heterogeneous and dynamic kinetics during cell fate transitions, highlighting its role in preserving transcriptome homeostasis. Conversely, disruption of RNA decay pathways has been implicated in developmental defects and disease, underscoring their potential as therapeutic targets. Collectively, RNA degradation emerges as a central regulator of PSC biology, integrating the decay of both mRNAs and ncRNAs to orchestrate pluripotency maintenance, lineage commitment, and disease susceptibility.

## 1. Introduction

Pluripotent stem cells (PSCs) possess the unique capacity for unlimited self-renewal and the ability to differentiate into all somatic lineages, making them indispensable models for early developmental biology and regenerative medicine [1,2]. While transcriptional regulation was long considered the main determinant of PSC identity, it is now evident that RNA turnover and stability are equally critical in orchestrating gene expression programs [3,4]. By dynamically regulating RNA lifespan, degradation pathways fine-tune transcript abundance and ensure the precise timing of developmental transitions [5,6].

In this review, we define RNA turnover as the dynamic equilibrium between RNA synthesis and degradation, reflecting the continuous renewal of RNA molecules within the transcriptome. In contrast, RNA decay specifically denotes the degradation process mediated by exonucleases, decapping complexes, RNA helicases, and the RNA exosome. This distinction is maintained throughout the manuscript to ensure terminological clarity and biological accuracy. The RNA degradation machinery is composed of multiple conserved pathways, including 5′–3′ exonucleolytic decay, deadenylation-dependent decay, nonsense-mediated decay, and mechanisms mediated by RNA-binding proteins or small RNAs [5]. Beyond protein-coding mRNAs, the decay of non-coding RNAs (ncRNAs), including long non-coding RNAs (lncRNAs), microRNAs (miRNAs), and circular RNAs (circRNAs) has emerged as a crucial regulatory layer that shapes gene networks and influences PSC fate by modulating transcript stability and signaling pathways [7].

In PSCs, these stability regulators intersect with epitranscriptomic modifications, particularly N6-methyladenosine (m^6^A), which can dictate transcript fate by promoting selective degradation [8,9]. Importantly, accumulating evidence indicates that RNA degradation and epigenetic landscapes are functionally interconnected [10]. Chromatin states and histone modifications can influence RNA metabolism, while RNA decay machinery reciprocally contributes to chromatin accessibility and enhancer activity, establishing feedback loops between transcriptional and post-transcriptional regulation [5,11,12]. Such multilayered networks suggest that RNA degradation is not a passive clearance mechanism but an active determinant of PSC identity. NcRNA decay follows similar principles, with the selective decay of specific lncRNAs and miRNAs reinforcing pluripotency or facilitating differentiation, illustrating the convergence of coding and non-coding RNA turnover in regulating PSC fate [13].

These mechanisms do more than support existing transcriptional programs, and instead they actively define developmental potential. Some studies emphasize the role of RNA degradation in clearing transcripts during lineage commitment, while others highlight its stabilizing influence on pluripotency networks through buffering transcriptional noise [5,14]. Together, these perspectives underscore the complexity of RNA stability regulation in PSCs and suggest that transcription, RNA decay, and epigenetic mechanisms converge within an integrated network that governs self-renewal and differentiation [15,16].

In this review, we address a critical gap in understanding RNA regulation in PSCs by integrating mRNA and ncRNA degradation with epitranscriptomic regulation. Whereas previous studies have focused on individual aspects of RNA decay or modifications, we synthesize current knowledge to show how RNA stability collectively shapes pluripotent state transitions. Specifically, we highlight (i) the coordinated action of core RNA decay machineries, (ii) the regulatory impact of post-transcriptional modifications and RNA-binding proteins, (iii) the dynamic remodeling and crosstalk of decay pathways during pluripotency and differentiation, and (iv) the broader implications of RNA stability control for development, disease, and therapeutic strategies. By offering this integrative perspective, we emphasize RNA degradation not merely as a quality-control mechanism but as a central regulator of PSC identity and lineage commitment.

## 2. Core mRNA Degradation Machinery

### 2.1. Exonucleolytic and Deadenylation-Dependent Decay Pathways

**Figure 1:**
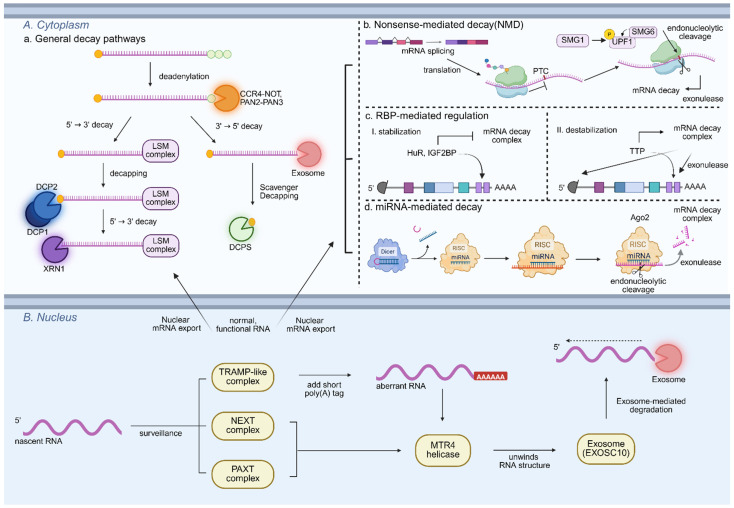
Overview of mRNA degradation pathways in pluripotent stem cells. (**A**) Cytoplasmic mRNA decay mechanisms. Cytoplasmic mRNA decay in PSCs proceeds through multiple co-existing pathways: a. General decay pathways, which involve deadenylation followed by either 5′→3′ exonucleolytic degradation by XRN1 or 3′→5′ degradation via the exosome; b. Nonsense-mediated decay (NMD), which selectively eliminates transcripts harboring premature termination codons (PTC); c. RNA-binding protein (RBP)-mediated decay, in which RBPs recognize cis-elements in target transcripts to modulate their stability or promote decay; d. miRNA-mediated decay, where the RNA-induced silencing complex (RISC) is guided by miRNAs to degrade or repress target mRNAs. (**B**) Nuclear RNA decay mechanisms. In the nucleus, improperly processed or aberrant RNAs are marked for degradation by the TRAMP-like complex in mammals, which consists of MTR4, PAPD5/7, and ZCCHC7/8. This complex adds short poly(A) tails and directs these RNAs to the nuclear exosome. Other surveillance pathways, such as the NEXT and PAXT complexes, recognize short-lived or polyadenylated nuclear RNAs without additional tagging and similarly channel them to the exosome via MTR4. In yeast, the functionally analogous machinery is known as the Trf4/5–Air2/1–Mtr4 polyadenylation (TRAMP) complex. The exosome, including its 3′→5′ exonuclease component EXOSC10 in mammals (RRP6 in yeast), facilitates RNA processing and decay to ensure transcriptome fidelity. RNAs that pass TRAMP-mediated quality control without errors are exported to the cytoplasm for translation. In the cytoplasm, RNA stability remains tightly regulated, with defective or improperly processed transcripts undergoing degradation through cytoplasmic decay pathways, thereby maintaining comprehensive transcriptome quality control. Created by Biorender.

Pluripotent stem cells (PSCs) maintain a highly dynamic transcriptome characterized by rapid RNA synthesis and turnover. This plasticity relies on canonical RNA degradation pathways (Figure 1, Table 1), particularly exonucleolytic and deadenylation-dependent decay [5].

In the nucleus, RNA surveillance and decay systems are tightly coordinated to eliminate aberrant or superfluous transcripts. The TRAMP-like complex adds short poly(A) tails to defective or unprocessed RNAs, thereby marking them for degradation by the nuclear exosome. In yeast, the TRAMP complex (Trf4/5–Air1/2–Mtr4) has been extensively characterized for this function [17,18]. In mammals, additional specialized RNA surveillance pathways complement this system. The Nuclear Exosome Targeting (NEXT) complex (ZCCHC8, RBM7, and MTR4) directs the exosome to short-lived non-coding RNAs such as PROMPTs and enhancer RNAs (eRNAs), whereas the Poly(A) tail Exosome Targeting (PAXT) connection, composed of ZFC3H1, MTR4, and PABPN1, targets polyadenylated nuclear RNAs, including improperly processed mRNAs, for exosome-mediated decay [19,20,21,22]. These nuclear RNA decay pathways are essential for maintaining transcriptomic integrity in PSCs, preventing the accumulation of non-functional or disruptive RNAs that could interfere with pluripotency maintenance, lineage priming, or stress response signaling [23,24].

In the cytoplasm, 5′→3′ exonucleases XRN1 and XRN2, together with the decapping machinery (DCP1/2 and the LSM1–7 complex), mediate transcript clearance, ensuring that pluripotency networks remain responsive to developmental and environmental cues [25]. Deadenylation complexes, including CCR4–NOT (Carbon Catabolite Repression 4 -Negative on TATA-less) and PAN2–PAN3 (Poly[A]-specific ribonuclease), further modulate stability by shortening poly (A) tails, a critical step that marks RNAs for subsequent degradation [26,27,28]. In addition to general RNA clearance, these pathways fine-tune transcript abundance and enable PSCs to swiftly adapt gene expression in response to differentiation signals or cellular stress [29]. Recent studies indicate that deadenylation-dependent decay is selectively regulated by RNA-binding proteins and post-transcriptional modifications, providing transcript-specific control that underlies cellular plasticity [26,30].

Complementing these mechanisms, the RNA exosome complex functions in both the nucleus and cytoplasm as a major 3′→5′ exonuclease, responsible for degrading misprocessed, misfolded, or surplus RNAs [31]. In embryonic stem cells (ESCs), the nuclear exosome cooperates with the TRAMP, NEXT, and PAXT pathways, while the cytoplasmic exosome operates alongside 5′→3′ decay and deadenylation mechanisms. This coordinated activity ensures comprehensive RNA surveillance and efficient transcript clearance during differentiation, underscoring the exosome’s central role in maintaining pluripotency [23,32,33]. Emerging evidence further suggests that exonucleolytic activity and substrate specificity are dynamically modulated by subcellular localization, post-transcriptional modifications, and cofactors. Together, these mechanisms establish a robust RNA surveillance network (Figure 1, Table 1) that balances transcriptome remodeling with the preservation of stem cell identity.

### 2.2. Nonsense-Mediated Decay and Quality Control Mechanisms

In addition to core exonucleolytic and deadenylation-dependent decay pathways, PSCs employ specialized quality-control mechanisms such as nonsense-mediated decay (NMD) to ensure transcript fidelity and regulate cell fate. NMD acts not only as a quality-control checkpoint but also as a regulatory pathway for PSC fate [34,35,36]. Core NMD components, including UPF1, SMG1, and SMG6, function in a coordinated manner (Figure 1, Table 1), where SMG1, a kinase, phosphorylates UPF1 to initiate NMD, and phosphorylated UPF1 then recruits SMG6, which mediates endonucleolytic cleavage of target transcripts, followed by further degradation of the resulting RNA fragments by 5′ → 3′ exonucleases such as XRN1 [37]. These components not only eliminate defective transcripts but also selectively degrade mRNAs encoding core pluripotency factors, positioning NMD as an active modulator of stem cell identity rather than a passive surveillance system. For instance, SMG6/UPF1-mediated NMD directly degrades c-Myc transcripts, thereby modulating self-renewal capacity and pluripotent state transitions in ESCs [14,38]. This regulatory specificity highlights how NMD contributes to the dynamic control of core transcriptional circuits and functions as a determinant of developmental potential.

Beyond pluripotency maintenance, NMD exerts lineage-specific functions during differentiation. In neural lineage commitment, NMD represses pluripotency-associated transcripts while stabilizing lineage-specific mRNAs, enabling a coordinated transcriptome switch [35,38]. This dual activity illustrates how NMD serves as both a safeguard and a dynamic regulator of developmental transitions [39]. Recent systems-level studies further suggest that NMD integrates with other decay pathways and post-transcriptional regulators to fine-tune transcriptome remodeling in response to developmental cues [40,41]. Collectively, these insights highlight NMD as a context-dependent regulator that bridges RNA surveillance with stem cell identity.

### 2.3. RNA-Binding Proteins and miRNA-Mediated Decay

Beyond these decay and quality-control pathways, transcript-specific regulation in PSCs is further refined by RNA-binding proteins (RBPs) and miRNA-mediated mechanisms [42,43,44]. RBPs such as HuR, IGF2BP, and TTP recognize specific sequence elements in target mRNAs, modulating their decay or stabilization in response to developmental cues [45,46]. In ESCs, HuR has been shown to bind and stabilize pluripotency-associated transcripts, thereby supporting stem cell identity and maintaining the undifferentiated state. In contrast, other RBPs, including TTP, selectively destabilize differentiation-related mRNAs by recognizing AU-rich elements (AREs) in the 3′ untranslated regions, promoting their endonucleolytic or exonucleolytic degradation, thereby preventing premature lineage commitment [42,47].

Complementing the action of RBPs, miRNAs offer an additional layer of post-transcriptional regulation in PSCs [48,49]. miRNAs are incorporated into the RNA-induced silencing complex (RISC), where Argonaute (AGO) proteins guide the complex to complementary mRNA targets and mediate their repression, with AGO2 uniquely capable of catalyzing endonucleolytic cleavage of the target RNA, followed by degradation of the resulting fragments by exonucleases such as XRN1 or the exosome complex [50,51]. The ESC-specific miR-290~295 cluster facilitates rapid G1–S transition by targeting cell cycle inhibitors, thereby enhancing both pluripotency and proliferation [52,53], while the miR-302 family fine-tunes lineage commitment by repressing transcripts that promote mesodermal and neural differentiation [54,55,56]. Collectively, RBP- and miRNA-mediated mechanisms form an integrated regulatory network tailored to PSCs, working in concert with canonical decay pathways to maintain transcriptome plasticity, reinforce pluripotency circuits, and enable timely responses to differentiation cues.

### 2.4. Compartmentalized RNA Surveillance in PSCs

PSCs preserve transcriptome integrity and adaptability through nuclear RNA surveillance and cytoplasmic RNA turnover [57]. In the nucleus, the RNA exosome, along with cofactors such as RRP6, MTR4, and the TRAMP complex, identifies and degrades aberrant RNAs, including mis-spliced or improperly processed transcripts, and thereby ensures the fidelity of splicing, 5′ capping, and 3′ end processing [58,59]. After nuclear export, mRNAs are subject to additional cytoplasmic decay via 5′→3′ exonuclease XRN1, 3′→5′ exosome-mediated decay, and decapping/deadenylation complexes. These canonical decay pathways are further modulated by RBPs and miRNA-associated silencing complexes, which confer transcript-specific control depending on developmental context [60,61].

This compartmentalized decay system ensures rapid clearance of pluripotency-associated transcripts and stabilization of lineage-specific mRNAs during differentiation, thereby enabling timely transitions in cell fate [38,62]. Nuclear surveillance and cytoplasmic turnover together function as integrated post-transcriptional mechanisms that maintain PSC identity and developmental plasticity. Although these processes have been primarily studied in mRNAs, growing evidence suggests that similar decay principles also apply to ncRNAs such as miRNAs, lncRNAs, and snoRNAs [63,64]. The pathways and regulatory factors involved in ncRNA decay are described in the following section, highlighting their distinct contributions to transcriptome remodeling and the regulation of pluripotent stem cell fate.

## 3. ncRNA Decay Pathways

PSCs utilize diverse ncRNAs, including miRNAs, lncRNAs, and small nucleolar RNAs (snoRNAs), as critical regulators of gene expression programs underlying self-renewal and lineage specification [44,65]. In contrast to protein-coding mRNAs, many ncRNAs are not translated and therefore largely escape translation-dependent quality control mechanisms such as NMD [66,67]. Instead, ncRNA degradation occurs through specialized decay pathways that operate independently of ribosomal engagement.

In the nucleus, the RNA exosome, in conjunction with cofactors such as MTR4 and the TRAMP complex, mediates the surveillance and degradation of aberrant or superfluous lncRNAs and snoRNAs, thereby preserving transcriptome integrity and preventing the accumulation of non-functional ncRNAs [68]. In the cytoplasm, ncRNA decay is further shaped by mechanisms such as target-directed miRNA degradation (TDMD) and RBP-mediated decay, which regulate the stability of mature miRNAs and lncRNAs in a context-dependent manner [69,70,71,72,73]. These cytoplasmic pathways play crucial roles in modulating the dynamics of pluripotency networks and lineage-specific gene expression.

Although ncRNA decay shares several conceptual features with mRNA degradation, such as reliance on RBPs, 3′ end trimming or deadenylation, and coordination between nuclear and cytoplasmic surveillance, it also exhibits distinct mechanistic characteristics, as summarized in Table 2. For example, while mRNAs are broadly regulated by NMD, XRN1-mediated 5′→3′ degradation, and deadenylation-coupled decay, ncRNAs are primarily targeted by nuclear exosome-dependent pathways (particularly for lncRNAs and snoRNAs) and non-canonical cytoplasmic mechanisms like TDMD, which function independently of translation initiation [66].

Together, these complementary mechanisms enable PSCs to dynamically regulate both coding and non-coding RNA populations, buffer transcriptional noise, and maintain the plasticity required for accurate cell fate transitions, thereby positioning ncRNA turnover as an integral component of post-transcriptional regulation that uniquely contributes to transcriptome remodeling and developmental potential in stem cell biology [74,75].

## 4. Epitranscriptomic Control of RNA Stability and Decay in PSCs

### 4.1. Regulation of RNA Stability by m^6^A, m^5^C, and Ψ in PSCs

PSCs employ a variety of chemical modifications on RNAs to regulate transcript stability and dynamically modulate gene expression programs critical for maintaining pluripotency and inducing differentiation [4,76]. Among these, N6-methyladenosine (m^6^A) is the most abundant internal mRNA modification, whereas 5-methylcytosine (m^5^C) and pseudouridine (ψ) exert distinct effects on RNA metabolism, influencing transcript stability, splicing, and translational control [77,78]. High-throughput techniques such as methylated RNA immunoprecipitation sequencing (MeRIP-seq), m^6^A-seq, 5′-bromo-uridine immunoprecipitation chase sequencing (BRIC-seq), and 4-thiouridine (4sU) labeling enable genome-wide mapping of RNA modifications and assessment of transcript stability and decay kinetics [79,80]. Importantly, not only mRNAs but also ncRNAs, such as miRNAs and lncRNAs, undergo chemical modifications including m^6^A, m^5^C, and ψ, which in turn influence their stability, processing, and regulatory potential in PSCs [44,81].

Accumulating evidence indicates that these RNA modifications often exhibit context-specific and functionally cooperative roles in determining PSC identity and facilitating lineage commitment [82]. For instance, m^6^A deposited by METTL3 promotes degradation of pluripotency-associated transcripts like *Nanog* and *Sox2* during early differentiation, ensuring timely exit from the pluripotent state. Conversely, loss of METTL3 leads to aberrant stabilization of these transcripts, ultimately delaying proper differentiation [9,83]. Likewise, the m^5^C methyltransferase NSUN2 stabilizes transcripts critical for neural differentiation, and its dysfunction leads to disrupted developmental gene expression programs [84,85]. Pseudouridylation, catalyzed by PUS family enzymes, enhances translational fidelity and stress adaptation, indirectly influencing transcriptome stability in PSCs [86,87]. These findings indicate that m^6^A primarily facilitates transcript decay to promote differentiation, whereas m^5^C and ψ contribute to transcript stability and cellular robustness, reflecting their distinct yet potentially cooperative roles in regulating pluripotent stem cell fate.

In addition, these chemical modifications extend beyond mRNAs to ncRNAs. For instance, m^6^A and other modifications on miRNAs regulate their biogenesis and decay, thereby adjusting the timing and magnitude of target repression during lineage specification. Pseudouridylation of lncRNAs can also influence their interactions with chromatin and RBPs, affecting epigenetic and transcriptional regulation [88,89]. RNA modifications represent dynamic and reversible regulatory marks deposited and removed by specific writer, reader, and eraser enzymes such as METTL3/14, FTO, ALKBH5, NSUN2, and PUS1. Notably, while m^6^A is reversible due to the presence of erasers such as FTO and ALKBH5, certain RNA modifications, including m^5^C and ψ, currently lack known erasers, rendering them post-transcriptionally irreversible. In the case of m^5^C, enzymatic oxidation produces derivatives such as hm^5^C or f^5^C rather than restoring unmodified cytosine, which contributes to RNA stability and functional robustness in pluripotent stem cells. These enzymes function in close coordination with RNA decay pathways to fine-tune transcript fate, linking the epitranscriptomic landscape to the regulation of RNA stability, decay, and functional dynamics in pluripotent stem cells [76,82]. This regulatory integration also applies to ncRNAs, where modification-dependent decay pathways interact with canonical RNA turnover mechanisms, including exosome-mediated surveillance and RBP- or miRNA-guided processes, to coordinately regulate the stability and dynamics of both coding and non-coding transcriptomes [90,91].

### 4.2. RNA-Binding Proteins as Modification Readers and Mediators of RNA Turnover

RBPs function as key readers of RNA modifications, interpreting chemical marks to direct downstream processes such as stability, localization, and degradation of transcripts [92,93,94]. For instance, the YT521-B Homology Domain Family (YTHDF) recognizes m^6^A-modified RNAs with functional specificity: YTHDF2 promotes mRNA decay through recruitment of the CCR4–NOT deadenylase complex, while YTHDF1 and YTHDF3 can enhance translation or cooperatively regulate decay depending on context [95,96,97]. Other RBPs, such as FMRP and HuR, participate in localization of modified RNAs to cytoplasmic granules like stress granules or P-bodies, thereby modulating transcript decay [98,99,100]. In PSCs, such RBPs coordinate both clearance of pluripotency transcripts and stabilization of lineage-specific mRNAs, contributing to proper timing of differentiation [42,62,101].

In addition to mRNAs, RBPs also recognize modified ncRNAs. For example, m^6^A-marked miRNAs and lncRNAs are bound by specific RBPs that influence their decay, stability, and interactions within RNA-protein complexes, thereby linking epitranscriptomic signals to broader non-coding RNA regulatory networks [102,103]. Many RBPs form regulatory hubs, interacting with each other and with decay machinery such as the exosome, XRN1/2, and miRNA-induced silencing complexes, integrating RNA modification signals with transcript clearance systems [104,105,106]. Experimental studies using RIP, iCLIP, or RBP perturbation models (e.g., knockdown or overexpression) have shown that RBPs play essential roles in shaping the PSC transcriptome in a differentiation-responsive manner [57,107,108]. These proteins serve as a bridge between epitranscriptomic cues and the RNA decay machinery, regulating both coding and non-coding RNA stability and ensuring precise transcriptomic remodeling in PSCs [15].

### 4.3. Epitranscriptomic Decay Dynamics Governing PSC Fate Transitions

RNA decay controlled by chemical modifications, a major component of epitranscriptomic regulation, is tightly linked to the transcriptomic plasticity of pluripotent stem cells PSC [76,92]. In particular, m^6^A-mediated decay targets key pluripotency factors such as *Oct4*, *Nanog*, and *Sox2* for degradation during the exit from the naïve state, while concurrently stabilizing lineage-associated transcripts to ensure proper fate transitions [109,110,111,112]. This regulation is executed by a network of m^6^A writers (METTL3/14), erasers (FTO, ALKBH5), and readers (YTHDF1–3), which collectively respond to developmental cues to modulate RNA fate [9,113,114].

**Figure 2:**
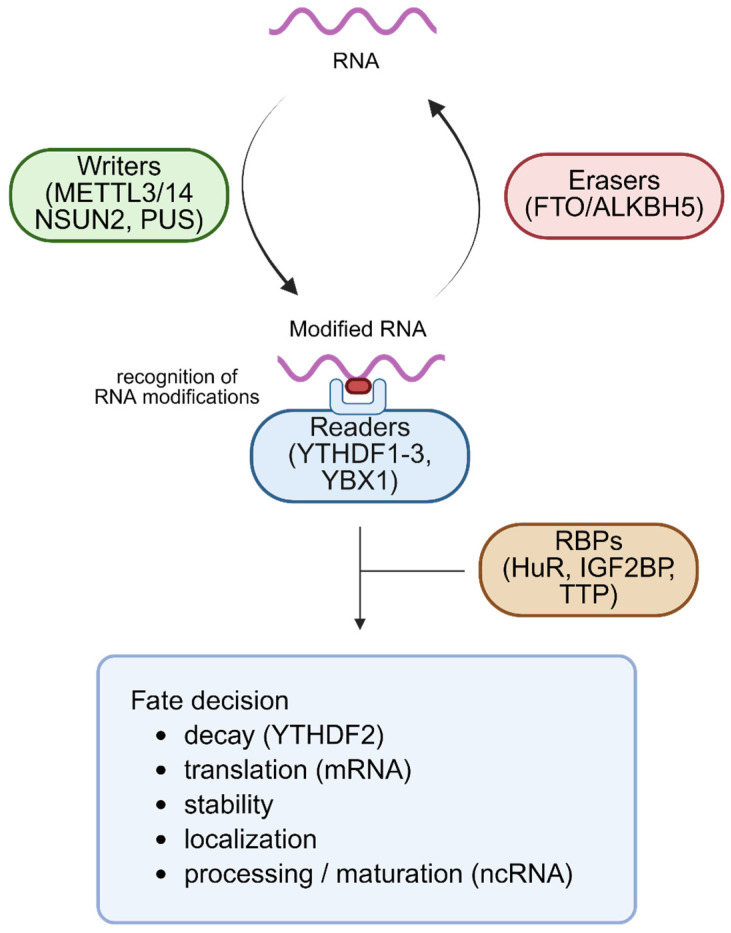
Epitranscriptomic regulation of coding and non-coding RNA fate in pluripotent stem cells. Chemical modifications on RNA molecules, including both mRNAs and ncRNAs, are dynamically regulated by writers (e.g., METTL3/14 for m^6^A, NSUN2 for m^5^C, PUS family for pseudouridylation), erasers (e.g., FTO, ALKBH5), and readers (e.g., YTHDF1–3, YBX1). These readers recognize specific epitranscriptomic marks to modulate key post-transcriptional processes such as RNA splicing, stability, localization, and translation. RBPs, including HuR, IGF2BP, and TTP, further influence the fate of both coding and non-coding RNAs by interacting with modified transcripts. Through the coordinated activity of these regulators, processes such as mRNA decay (e.g., YTHDF2-mediated), translational control, transcript stabilization, and ncRNA maturation are precisely orchestrated. This integrated regulatory network shapes pluripotent stem cell identity and enables dynamic gene expression programs during cell fate transitions. Created by Biorender.

Beyond mRNAs, m^6^A also modifies ncRNAs, including miRNAs and lncRNAs, influencing their processing, decay, and activity. These modifications shape post-transcriptional gene regulatory networks that are critical for maintaining or dissolving pluripotency [115]. Functional studies have shown that disruption of this regulatory layer, such as through METTL3 knockdown or depletion of YTHDF proteins, results in delayed differentiation and impaired lineage specification, underscoring the pivotal role of epitranscriptomic RNA decay in PSC biology [110,112,116]. Simultaneously, m^6^A marks guide miRNA maturation, reinforce miRNA-mediated repression, and regulate lncRNA stability, operating in concert with canonical decay pathways to ensure balanced control of coding and non-coding transcriptomes [117]. A schematic overview of RNA modifications, their recognition by RBPs, and their integration with RNA decay pathways in PSCs is illustrated in Figure 2, and Table 3 summarizes the key writer, reader, and eraser proteins and their roles in stem cell fate regulation.

Finally, m^6^A-regulated decay cooperates with core degradation machineries, including the exosome, XRN1/XRN2 exonucleases, and deadenylation complexes, and works in parallel with miRNA-guided silencing to maintain transcriptome homeostasis [118,119]. In addition to these cooperative pathways, spatial compartmentalization between the nucleus and cytoplasm further contributes to the precise and context-dependent control of RNA turnover during PSC state transitions [120,121,122].

This compartmentalized regulation similarly applies to ncRNAs, allowing spatially controlled decay of lncRNAs and miRNAs, which supports precise timing of lineage-specific gene expression and sustains the plasticity of the PSC transcriptome [123]. High-throughput approaches such as MeRIP-seq combined with BRIC-seq or 4sU labeling have been instrumental in mapping these dynamics and establishing m^6^A-mediated decay as a pivotal driver of PSC transcriptome plasticity, enabling rapid and precise cell fate transitions in response to developmental and environmental cues [80,124]. Collectively, these findings reveal that chemical modifications act through an integrated epitranscriptomic decay network encompassing both coding and non-coding RNAs, thereby orchestrating dynamic transcriptome remodeling in PSCs.

## 5. Dynamic Remodeling and Crosstalk of RNA Decay Pathways

### 5.1. RNA Decay Dynamics During State Transitions

PSCs undergo extensive remodeling of RNA turnover as they transition between distinct pluripotent states and during lineage commitment [3,125,126]. Naïve and primed PSCs exhibit distinct transcriptome dynamics, with naïve cells generally showing elevated RNA synthesis and accelerated decay rates, thereby facilitating rapid responsiveness to developmental cues [127,128]. During somatic cell reprogramming, RNA decay pathways are restructured to erase somatic transcriptional memory and to establish pluripotency-associated gene expression profiles [5,129]. Similarly, during directed differentiation, selective stabilization of lineage-specific mRNAs and coordinated clearance of pluripotency-associated transcripts underscore the importance of tightly regulated transcript turnover in cell fate transitions [43,76,130].

**Figure 3:**
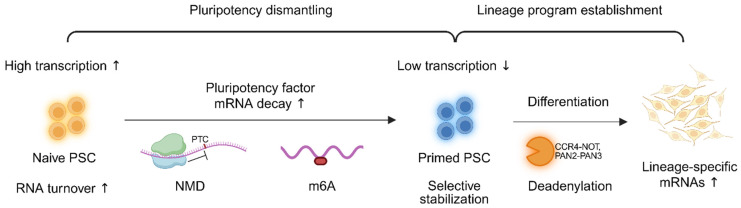
RNA decay dynamics governing the transition from naïve to primed pluripotent states. During the naïve-to-primed transition, NMD and m^6^A-mediated decay selectively remove pluripotency-associated transcripts, while deadenylation-dependent pathways clear lineage-specific transcripts in primed PSCs. This coordinated reprogramming of RNA decay pathways highlights a novel post-transcriptional mechanism that regulates pluripotent stem cell fate during state transitions. Upward arrows (↑) indicate increased levels, while downward arrows (↓) indicate decreased levels. Created by Biorender.

Recent findings highlight that distinct RNA decay mechanisms differentially influence cell fate. For instance, NMD preferentially destabilizes pluripotency regulators, whereas deadenylation complexes play a more prominent role in removing transcripts induced during differentiation [37]. Epitranscriptomic modifications, particularly m^6^A, also contribute to the fine-tuning of decay kinetics. For example, METTL3-mediated methylation promotes the degradation of pluripotency-related mRNAs during the transition from the naïve to the primed state [112,131,132]. These decay pathways and their remodeling across stem cell states are summarized in Figure 3 and Table 4, which highlight key regulators, target transcripts, and functional implications for pluripotency exit and lineage commitment.

Advances in single-cell RNA sequencing (scRNA-seq) combined with RNA velocity analyses have revealed that decay rates are not uniform but vary across differentiation trajectories. For example, neural and mesendodermal lineages exhibit distinct decay kinetics, reflecting pathway-specific remodeling of RNA decay during early fate specification [133]. Additionally, external signals such as WNT and FGF rapidly modulate RNA decay rates, providing a fast-acting post-transcriptional layer that complements transcriptional responses [35,134,135]. While most studies have focused on mRNA dynamics, recent evidence suggests that ncRNAs (including lncRNAs, circRNAs, and miRNAs) also undergo precisely regulated turnover in PSCs, which contributes to the maintenance of pluripotency and the regulation of lineage commitment [43].

### 5.2. Functional Impact of Non-Coding RNA Turnover in PSCs

Beyond mRNA, ncRNAs, including miRNAs, lncRNAs, and circRNAs, are subject to active regulation by RNA decay pathways in PSCs [44,65,136]. miRNA turnover shapes post-transcriptional repression networks, whereas lncRNA and circRNA stability affects gene regulation, RNA–chromatin interactions, and enhancer activity [137,138,139]. Dysregulated ncRNA decay has been implicated in defective neurogenesis and broader developmental abnormalities, emphasizing its functional importance [140,141,142]. For instance, altered miRNA stability can disrupt the delicate balance between self-renewal and differentiation, while precise lncRNA decay is essential for accurate lineage specification [143,144].

Emerging studies reveal the mechanistic diversity of ncRNA degradation. TDMD actively regulates the abundance of key miRNA families such as the miR-290/302 cluster and let-7, thereby modulating the balance between self-renewal and differentiation states [145]. Likewise, the degradation of pluripotency-linked lncRNAs such as lincRNA-RoR and TUNA is coordinated by the exosome or 5′→3′ exonucleases like XRN1/XRN2, influencing enhancer activity and chromatin accessibility [146,147]. Although circRNAs are generally more stable than their linear counterparts, their decay is governed by specific ribonucleases and modulated by miRNA sponge dynamics, and circRNA remodeling has been observed during PSC differentiation [148,149]. Dysregulation of these decay mechanisms has been linked to aberrant chromatin architecture, improper lineage specification, and neurodevelopmental disorders [150,151,152]. Together, these findings position ncRNA turnover as a key regulatory layer that complements coding RNA decay to define PSC identity and drive cell fate transitions. This interconnected degradation of coding and non-coding transcripts forms the foundation of the integrated RNA decay network discussed below.

### 5.3. Crosstalk and Integration of Core Decay Pathways

RNA decay in PSCs operates through a highly interconnected network rather than isolated mechanisms, encompassing NMD, Staufen-Mediated mRNA Decay (SMD), the exosome complex, and cytoplasmic decay machineries to ensure transcript quality control and abundance regulation [5,14,29]. This network is further modulated by RBPs and miRNA-mediated silencing, which confer transcript specificity and integrate epitranscriptomic signals such as m^6^A, m^5^C, and ψ to guide RNA stability [153,154]. Subcellular compartmentalization adds additional complexity, as nuclear and cytoplasmic decay pathways, along with specialized structures such as P-bodies and stress granules, influence the selection and fate of target RNAs [61,155]. Ribosome profiling studies show that translation and decay are intimately coupled, with ribosome stalling or occupancy often guiding the recruitment of decay factors [156].

Beyond these molecular interactions, RNA decay pathways engage in adaptive feedback loops that influence pluripotency gene networks and signaling cascades [157]. Throughout PSC state transitions, including reprogramming and lineage differentiation, the remodeling of decay machinery enables rapid and flexible adaptation of the transcriptome [158,159]. Mechanistic crosstalk among decay pathways can be cooperative or competitive, exerting overlapping or synergistic control over shared transcript targets, thereby ensuring precise and context-dependent regulation of gene expression during pluripotency maintenance and differentiation [160,161]. Systems-level modeling and perturbation analyses have begun to unravel the architectural logic of these decay networks, revealing how transcriptome homeostasis and cell identity are safeguarded by interconnected RNA decay modules [162]. This coordinated crosstalk among RNA decay pathways constitutes a systems-level regulatory framework essential for preserving stem cell identity and enabling flexible transcriptomic adaptation during differentiation. Importantly, this integrated framework not only underpins normal development but also offers insights for regenerative medicine, where manipulating RNA decay could enhance reprogramming efficiency, promote lineage conversion, or correct pathological transcriptome imbalances.

## 6. Technological Advances and Clinical Implications

Building upon the mechanistic insights described above, recent advances in high-throughput technologies have enabled precise quantification and systems-level integration of RNA decay processes in PSCs. These tools have expanded our understanding of how RNA turnover contributes to cellular heterogeneity, lineage commitment, and disease progression.

### 6.1. Single-Cell and Multi-Omics Approaches

Recent advances in scRNA-seq have enabled high-resolution dissection of transcriptome dynamics, revealing substantial heterogeneity in RNA decay among individual PSCs, even within phenotypically homogeneous populations [163,164]. These mechanistic frameworks are now being interrogated with unprecedented resolution through advanced single-cell and multi-omics platforms, revealing deeper insights into the dynamics and regulation of RNA decay. These analyses have uncovered subpopulations with distinct decay kinetics, illustrating that transcript-specific stability plays a key role in buffering transcriptional noise and maintaining pluripotency [165]. Furthermore, metabolic labeling approaches at the single-cell level, such as single-cell thiol(SH)-Linked Alkylation for the Metabolic sequencing of RNA (scSLAM-seq), enable direct measurement of RNA synthesis and degradation rates by distinguishing newly transcribed RNA from pre-existing molecules [166,167]. These methodologies demonstrate that RNA turnover is dynamically regulated depending on cell state, developmental trajectory, and extrinsic signals, with specific decay pathways targeting subsets of transcripts in a context-dependent manner.

Integrating single-cell transcriptomics with complementary omics layers, such as chromatin accessibility (scATAC-seq) and RNA modification profiling, has enabled a systems-level understanding of RNA decay regulation [168]. Multi-omics approaches have revealed that RNA stability is tightly coordinated with epigenetic states and post-transcriptional modifications. These findings suggest that dynamic decay processes not only buffer cellular heterogeneity but also actively regulate stem cell plasticity, lineage commitment, and responsiveness to developmental cues [169,170]. Importantly, perturbations in RNA decay pathways can disrupt differentiation timing, compromise lineage fidelity, and impair developmental progression, underscoring the biological significance of regulated RNA turnover in early embryogenesis [165]. Collectively, single-cell and multi-omics analyses position RNA decay as a central mechanism governing transcriptome remodeling and fate decisions in pluripotent systems.

### 6.2. Dysregulation in Disease and Therapeutic Opportunities

Genetic alterations in RNA decay pathways have been implicated in diverse human disorders, including amyotrophic lateral sclerosis (ALS), frontotemporal dementia (FTD), and spinal muscular atrophy (SMA) [171,172]. Mutations in core components of the decay machinery, such as UPF1, SMN, and exosome subunits, can result in aberrant accumulation or insufficient degradation of specific transcripts, disrupting gene expression homeostasis, particularly in neurons and other vulnerable cell types [173,174]. Dysregulated RNA turnover is not limited to adult-onset neurodegeneration but also impacts PSCs and early embryonic development, where improper clearance or stabilization of pluripotency- or lineage-specific transcripts can compromise stem cell identity, disrupt differentiation, and impair developmental progression [4,5].

Beyond disease mechanisms, RNA decay pathways are increasingly recognized as viable therapeutic targets. Strategies such as antisense oligonucleotides (ASOs), RNA interference (RNAi), and small-molecule modulators enable selective manipulation of transcript stability [175,176]. For example, ASO-mediated correction of SMN2 splicing has successfully restored SMN protein expression in SMA patients, validating the clinical relevance of RNA decay modulation [177,178]. Similarly, RNAi and small molecules are being developed to enhance the degradation of toxic RNAs in neurodegenerative conditions such as ALS and FTD, offering promising avenues for precision medicine [179,180].

In regenerative medicine, insights into RNA decay dynamics hold potential for improving stem cell–based therapies. Fine-tuning the stability of pluripotency- and lineage-specific transcripts can enhance the efficiency of reprogramming, lineage commitment, and functional maturation of differentiated cells [129,181]. Understanding how RNA turnover regulates transcriptional plasticity and facilitates cell state transitions is increasingly recognized as a key aspect of developmental biology and an essential basis for improving regenerative and therapeutic strategies [125]. Taken together, these findings underscore RNA decay as a pivotal regulator of cellular homeostasis and a promising target for both disease intervention and regenerative applications.

## 7. Conclusions

RNA degradation is now recognized not merely as a passive quality control mechanism but as a dynamic regulatory force essential for maintaining PSC identity and guiding cell fate decisions. By interacting with epitranscriptomic modifications and chromatin-based mechanisms, RNA turnover forms an integral layer of post-transcriptional regulation that modulates gene expression networks, sustains pluripotency, and orchestrates timely lineage commitment [76,182].

This review has underscored the multifaceted roles of RNA decay, its crosstalk with epigenetic processes, and its broader implications for development and disease. Collectively, these insights position RNA degradation as a central hub in the regulatory architecture of stem cell biology. Advancing this field will require not only deeper mechanistic understanding of degradation pathways but also their contextual integration into epigenetic, transcriptomic, and systems-level regulatory frameworks. Moving forward, integrating RNA decay analyses with spatial transcriptomics and synthetic regulatory systems may unlock new strategies for controlling stem cell fate and designing targeted regenerative therapies.

## 8. Future Directions

Building on these conclusions, future investigations RNA degradation in PSCs should focus on three critical areas: (I) unresolved mechanistic questions, such as transcript-selective targeting, spatiotemporal regulation, and coordination with translation; (II) the dynamics and regulatory roles of ncRNA turnover, including lncRNAs, circRNAs, and miRNAs; and (III) the application of emerging technologies and systems-level approaches to model RNA decay networks and identify novel therapeutic opportunities.

Despite substantial progress, many fundamental questions remain. These include how degradation pathways achieve transcript-specific selectivity, how RNA turnover is spatially and temporally regulated across nuclear and cytoplasmic compartments, and how these processes intersect with translational control. Resolving these questions is essential to fully understand the role of RNA stability in maintaining pluripotency and directing lineage commitment.

The turnover of ncRNAs remains a particularly underexplored area. While lncRNAs, circRNAs, and miRNAs are known to modulate gene expression, chromatin accessibility, and signaling cascades, their degradation dynamics in PSCs are only beginning to be defined [183]. Elucidating how the decay of coding and non-coding transcripts is coordinated will offer a more integrated view of transcriptome regulation. To provide a concrete overview of how different RNA decay pathways contribute to PSC functions and differentiation states, we summarize key pathways, their target gene groups, and associated effects in Table 5.

Recent advances in RNA technologies provide powerful tools to interrogate RNA degradation with unprecedented resolution. Approaches such as single-cell multi-omics, scSLAM-seq, RNA velocity, and long-read sequencing now allow simultaneous profiling of transcript abundance, RNA modifications, splicing isoforms, and chromatin states. Applying these technologies will enable precise dissection of RNA decay kinetics in a cell-state- and lineage-specific context, uncovering heterogeneity and dynamic remodeling previously masked in bulk analyses. For example, (i) combining scSLAM-seq with CRISPRi-mediated knockdown of specific RNA decay factors could reveal causal effects on transcript-specific stability in PSCs; (ii) metabolic RNA labeling with 4sU or EU followed by long-read sequencing could quantify half-lives of coding and non-coding transcripts, including lncRNAs and circRNAs, at isoform resolution; and (iii) live-cell imaging of RNA decay reporters would allow direct visualization of the spatial and temporal dynamics of transcript turnover. Integration of multi-omics data with computational modeling and network analysis will facilitate the identification of key regulatory nodes, pathway crosstalk, and feedback loops that govern PSC plasticity and fate transitions.

Finally, manipulating RNA stability holds considerable promise for therapeutic development. Targeting RNA decay pathways using small molecules, ASOs, or RNAi could restore transcriptome balance, improve reprogramming efficiency, and guide lineage specification in regenerative medicine. PSC-based models also offer a robust platform to examine how RNA decay impacts developmental disorders, neurodegeneration, and cancer. Collectively, these strategies highlight RNA degradation not only as a fundamental regulator of stem cell biology but also as a versatile axis for therapeutic intervention.

## Figures and Tables

**Table 1:** Major Pathways and Key Enzymes in RNA Degradation.

Step	Key Complex/Enzyme	Direction	Description
Deadenylation	CCR4-NOT, PAN2-PAN3	3′ → 5′	Shortening of the poly (A) tail
Decapping	DCP1/DCP2	5′ → 3′	Removal of the 5′ cap
5′ → 3′ decay	XRN1	5′ → 3′	Degradation following decapping
3′ → 5′ decay	Exosome	3′ → 5′	Degradation after poly (A) tail removal
Nonsense-mediated decay	UPF1, SMG1, SMG6	Specialized	Elimination of mRNAs with premature stop codons
miRNA-mediated decay	RISC (Ago2, TRBP, Dicer), miR-290~295, miR-302	Specialized	Selective degradation of target mRNAs
RBP-mediated regulation	HuR, IGF2BP, TTP	Specialized	Stabilization or degradation of target mRNAs depending on the RBP

**Table 2:** Comparison of mRNA and ncRNA decay pathways in pluripotent stem cells.

Feature/Pathway	mRNA Decay	ncRNA Decay	Common Features
Nuclear decay	Exosome, TRAMP, Nuclear surveillance	Exosome, TRAMP (mainly lncRNA, snoRNA)	Exosome-mediated RNA quality control
Cytoplasmic decay	5′ → 3′ XRN1, NMD, deadenylation, RBP/miRNA-mediated	TDMD, RBP-mediated, some lncRNAs via exosome	RBP-dependent regulation, decapping/deadenylation involvement
NMD	Applied	Mostly not applied	Rarely engaged in ncRNAs
miRNA-related	miRNAs regulate mRNA targets	miRNA turnover (TDMD)	miRNA-associated RBP involvement
Function	Transcriptome remodeling, pluripotency and differentiation control at mRNA level	Gene regulation, fine-tuning pluripotency networks, differentiation control	RNA turnover modulates PSC fate decisions

**Table 3:** RNA Modifications, Associated Enzymes, and Functional Impact in PSCs.

Modification	Writer(s)	Reader(s)	Eraser(s)	Function & PSC Fate Impact
m^6^A	METTL3/14	YTHDF1/2/3 IGF2BP	FTO, ALKBH5	- mRNA stability, decay, translation, nuclear export - lncRNA nuclear retention, chromatin silencing - pri-miRNA processing, snRNA splicing - PSC fate: pluripotency maintenance and differentiation transition control
m^5^C	NSUN2/6, DNMT2	ALYREF, YBX1	-	- mRNA stability, translation efficiency enhancement, cellular stress response - tRNA stabilization, rRNA translation fidelity assurance - lncRNA export, subcellular localization - PSC fate: stress adaptation, neural differentiation, transcriptome plasticity maintenance
ψ (pseudouridine)	PUS family	Ribosome, spliceosome machinery	-	- Translation accuracy enhancement, stop codon readthrough, stability - rRNA/snRNA processing fidelity assurance - tRNA structure stabilization - lncRNA translation regulation - PSC fate: ESC differentiation program modulation, translational plasticity maintenance

**Table 4:** Regulation of transcript turnover in naïve-to-primed differentiation.

Feature	Naïve PSC	Primed PSC
Global transcription rate	High	Moderate
RNA decay speed	Fast	Moderate
NMD target	Pluripotency factors	Lineage-specific mRNAs
Deadenylation activity	Low	High
m^6^A-mediated decay	Accelerated	Selective stabilization

**Table 5:** RNA decay pathways and their contributions to PSC functions and differentiation states.

RNA Decay Pathway	Key Target Genes/RNAs	Cellular Location	PSC Function/Differentiation Pathway	Effect on PSC State
NMD	*Oct4*, *Sox2*, PTC-containing mRNAs, etc.	Cytoplasm	Pluripotency maintenance	- Prevents accumulation of aberrant transcripts - supports self-renewal
miRNA-mediated decay	miR-290/302 targets, *Lin28a*, *Lin28b*, etc.	Cytoplasm, P-bodies	Pluripotency & lineage bias	- Fine-tunes pluripotency - directs lineage specification
CCR4-NOT deadenylation complex	Cyclin mRNAs, pluripotency TFs, etc.	Cytoplasm	Cell cycle control, pluripotency	- Shortens poly(A) tails → transcript degradation, regulates self-renewal
Exosome-mediated 3′ → 5′ decay	PROMPTs, unstable nuclear RNAs, etc.	Nucleus/ Cytoplasm	RNA quality control, pluripotency maintenance	- Removes aberrant or non-functional RNAs - maintaining transcriptome integrity
RBP-mediated decay	Differentiation-related TFs, signaling modulators, etc.	Cytoplasm	Lineage specification	- Post-transcriptionally regulates mRNA stability to control differentiation timing

## Data Availability

No new data were created or analyzed in this study.

## Abbreviations

PSC	Pluripotency Stem Cell
TRAMP	Trf4/5–Air1/2–Mtr4 Polyadenylation Complex
NEXT	Nuclear Exosome Targeting Complex
PAXT	Poly(A) tail Exosome Targeting Complex
PROMPTs	Promoter Upstream Transcripts
NMD	Nonsense-Mediated Decay
TDMD	Target-Directed miRNA Degradation
RBP	RNA-Binding Protein
m^6^A	N6-Methyladenosine
m^5^C	5-Methylcytocine
TTP	Tristetraprolin
ARE	AU-Rich Element
RISC	RNA-Induced Silencing Complex
AGO	Argonaute Protein
WNT	Wingless/Integrated Signaling Pathway
FGF	Fibroblast Growth Factor
SMD	Staufen-Mediated mRNA Decay
ALS	Amyotrophic Lateral Sclerosis
FTD	Frontotemporal Dementia
SMA	Spinal Muscular Atrophy
ASO	Antisense Oligonucleotide
